# Methodological comparison of fetal brain ^1^H‐MRS data analysis techniques and gestational age analysis using LCModel in the fetal sheep brain

**DOI:** 10.1113/EP093728

**Published:** 2026-03-02

**Authors:** Jordan A. Minns, Jack R. T. Darby, Georgia K. Williams, Jesse T. Tu, Ziqi Sun, Jessie Guo, Steven P. Miller, Christopher K. Macgowan, Mike Seed, Jamie Near, Janna L. Morrison

**Affiliations:** ^1^ Early Origins of Adult Health Research Group, Robinson Research Institute, School of Pharmacy and Biomedical Sciences Adelaide University Adelaide South Australia Australia; ^2^ Preclinical, Imaging and Research Laboratories South Australian Health and Medical Research Institute Adelaide South Australia Australia; ^3^ Translational Medicine Hospital for Sick Children Toronto Ontario Canada; ^4^ University of British Columbia Vancouver British Columbia Canada; ^5^ British Columbia Children's Hospital Research Institute Vancouver British Columbia Canada; ^6^ Department of Medical Biophysics University of Toronto Toronto Ontario Canada; ^7^ Division of Cardiology Hospital for Sick Children Toronto Ontario Canada; ^8^ Department of Paediatrics University of Toronto Toronto Ontario Canada; ^9^ Sunnybrook Research Institute, Physical Sciences Platform Hurvitz Brain Sciences Program Toronto Ontario Canada; ^10^ Department of Physiology, Faculty of Medicine University of Toronto Toronto Canada

**Keywords:** fetus, neurodevelopment, normative metabolite reference, sheep, spectroscopy

## Abstract

Proton (^1^H) magnetic resonance spectroscopy (MRS) is a non‐invasive imaging technique that can be used to assess brain metabolism. Establishing reference levels of fetal brain metabolites in a translatable preclinical model is crucial for identifying subtle deviations from normal cerebral development. Herein, we developed a ^1^H‐MRS protocol for fetal sheep across three gestational periods (109 ± 2, 120 ± 5 and 139 ± 2 days gestation; term is 150 days gestation). Using this protocol, we established reference metabolic levels using recommended ^1^H‐MRS data analysis techniques. Objectives included adapting protocols for reliable metabolite detection despite the challenges of fetal ^1^H‐MRS, applying the protocol at multiple gestational ages, using recommended processing techniques and comparing two analysis software packages for robustness. MRI scans were performed on a 3 T Siemens clinical system (Magnetom Skyra, Siemens Healthineers, Germany) while the ewe was ventilated. ^1^H‐MRS was performed using a point‐resolved spectroscopy sequence at an echo time of 135 ms and a voxel size of 15 mm × 15 mm × 15 mm, gated to maternal respiration. We showed that TARQUIN metabolite level estimates had a significantly higher Cramér–Rao lower bound uncertainty compared with LCModel in *N*‐acetyl aspartate and choline, with differences in fit quality, while also underestimating metabolites. LCModel was then used to determine reference levels for *N*‐acetyl aspartate, choline, creatine and lactate across three gestational time points in late gestation. Our findings demonstrate the feasibility and reproducibility of non‐invasive fetal sheep brain ^1^H‐MRS. The study highlights the importance of objective criteria for accurate data interpretation, providing insights into fetal brain development and potential non‐invasive applications for earlier detection of poor neurodevelopmental outcomes.

## Abstract (highlights)

- **What is the central question of this study?**
  Can proton magnetic resonance spectroscopy (^1^H‐MRS) be used reliably to assess cerebral metabolism non‐invasively in a preclinical fetal sheep model throughout gestation, and what is the most robust analysis method for fetal sheep data?
- **What is the main finding and its importance?**
  This study established a preclinical fetal sheep ^1^H‐MRS protocol capable of measuring cerebral metabolites non‐invasively. LCModel was shown to provide more robust fetal data analysis than TARQUIN, and reference metabolite levels were defined throughout late gestation. These findings support the use of ^1^H‐MRS as a non‐invasive tool for assessing fetal brain metabolism in preclinical models.

## 1 INTRODUCTION

Proton (^1^H) magnetic resonance spectroscopy (MRS) is a technique that allows non‐invasive in vivo metabolite quantification and can provide valuable insights into fetal brain biochemistry and pathophysiological mechanisms (Berger‐Kulemann et al., 2013; Faghihi et al., 2017; Pugash et al., 2009). Unlike traditional MRI, such as T_1_‐ and T_2_‐weighted techniques, ^1^H‐MRS can detect metabolic changes that may precede structural abnormalities (Berger‐Kulemann et al., 2013; Pugash et al., 2009). This technique leverages the magnetic properties of hydrogen (^1^H) to acquire a spectrum through the chemical shift phenomenon, allowing for the identification of chemical compounds (Fujita, 2007).

During fetal brain development, neurons and neuronal connections are formed. ^1^H‐MRS allows for the quantification of specific metabolites, such as: *N*‐acetyl aspartate (NAA), a marker of neuronal development and viability; creatine (Cr), a marker of cellular energy metabolism; choline (Cho), a marker of cell membrane turnover; and lactate, a fuel source in the developing fetal brain and by‐product of glycolysis (Pradhan et al., 2020). However, acquiring high‐quality spectra consistently from the fetal brain is challenging owing to factors such as maternal and fetal motion, fetal positioning and low signal attributable to amniotic fluid and maternal organs (Pradhan et al., 2020; Story et al., 2011). As a result of these challenges, few have characterized the normal biochemical evolution of these key metabolites across gestation (Berger‐Kulemann et al., 2013; Pradhan et al., 2020). Instead, a higher degree of focus has been on the evolving biochemical profiles observed *ex utero* in preterm infants, neonates and children (Bluml et al., 2013; Horska et al., 2002; Hyodo et al., 2018; Kadota et al., 2001; Kreis et al., 1993).

When quantifying metabolite levels in ^1^H‐MRS, factors influencing the magnetic resonance (MR) signal properties, such as the number of equivalent protons and the specific acquisition method used, must be taken into account. Only metabolites that are freely tumbling and not bound in myelin or membranes are generally detectable using ^1^H‐MRS (Girard et al., 2006c; Story et al., 2011). To obtain estimates of metabolite levels, signals must be compared with a reference signal with known levels, ideally from a stable compound in the spectrum, such as tissue water (Story et al., 2011). These factors have limited the successful implementation of in vivo fetal ^1^H‐MRS in both clinical and research settings. Generally, metabolite ratios rather than absolute metabolite levels have been reported, because absolute metabolite levels can vary owing to technical factors, such as voxel placement and tissue composition (Story et al., 2011). Hence, establishing reference levels of these metabolites is crucial for identifying subtle but important deviations from normal development in high‐risk fetuses, offering more informed surveillance during pregnancy and pregnancy research. The reliability of ^1^H‐MRS is typically assessed using Cramer–Rao lower bounds (CRLB), which indicate the uncertainty or variance in the estimated metabolite concentrations (Pradhan et al., 2020). However, there has been concern around the use of this threshold to exclude spectra (Kreis, 2016). This is particularly relevant in the developing fetal sheep brain, which remains smaller than the human brain even in late gestation; as a result, metabolite signals are inherently low and more challenging to quantify, with literature on the recommended processing and analysis approaches being limited.

Thus, the aim of this study was to develop a fetal sheep ^1^H‐MRS protocol to measure brain metabolites across three gestational periods, establishing normal metabolite levels and their developmental trajectories. Our objectives included establishing a protocol for accurate metabolite detection despite the unique challenges of fetal ^1^H‐MRS, applying this protocol across multiple gestational ages, using recommended ^1^H‐MRS processing techniques to analyse the spectral data and comparing two currently available analysis software packages to recommend the more effective method for future use.

## 2 MATERIALS AND METHODS

### 2.1 Ethical approval

Animal ethics approval was obtained from the South Australian Health and Medical Research Institute's Animal Ethics Committee (SAM‐389.19) for all research described herein and followed the Australian Code of Practice for the Care and Use of Animals for Scientific Purposes. The experiment was designed in line with the ARRIVE guidelines (Kilkenny et al., 2010; Percie du Sert et al., 2020). Ewes from the South Asutralian Health and Medical Research Institute's farm (Burra, South Australia) were housed in an indoor facility with a constant temperature of 20°C–22°C and a 12 h–12 h light–dark cycle. Each ewe was housed in an individual pen within view of other ewes, with ad libitum access to water and food. All investigators were aware of the ethical principles outlined by *Experimental Physiology* (Grundy, 2015; O'Halloran, 2024) along with the principles of the 3Rs, especially the reduction of animal use within scientific research (Lindsjo et al., 2016).

### 2.2 Animals and surgery

At 116–124 days gestational age (dGA; term, 150 dGA), Merino ewes (*n* = 39) carrying 36 singletons and 3 twins for a total of 42 fetuses (male, *n* = 22; female, *n* = 20) underwent surgery in aseptic conditions, as previously described (Edwards et al., 1999; Morrison et al., 2001). Anaesthesia was induced with intravenous diazepam (0.3 mg/kg) and ketamine (5 mg/kg) and maintained with inspired isoflurane (1.5%–2.5% in 100% oxygen). The depth of anaesthesia appropriate for the commencement of surgical procedures was determined via absence of the eye reflex. Vascular catheters were filled with heparinized saline (0.9% NaCl) and implanted into the maternal jugular vein, fetal femoral vein, umbilical vein, femoral artery and the amniotic cavity as previously described (Darby et al., 2020; Edwards et al., 1999; Morrison et al., 2001). The uterus was then closed in two watertight layers around the catheters, and the catheters were tunnelled through an incision in the flank of the ewe. Catheters were flushed daily with heparinized saline (0.9% NaCl) to ensure that they did not become obstructed.

Ewes received an intramuscular injection of antibiotics [3.5 mL of Duplocillin (150 mg/ml procaine penicillin and 112.5 mg/mL benzathine penicillin; Norbrook Laboratories Ltd, Gisborne, VIC, Australia)] and 2 mL of 125 mg/mL dihydrostreptomycin (Sigma‐Aldrich, St Louis, MO, USA) at surgery and daily for 3 days postoperatively. Fetuses received an intramuscular injection of 1 mL of Duplocillin (150 mg/mL procaine penicillin and 112.5 mg/mL benzathine penicillin) and 1 mL of 125 mg/mL dihydrostreptomycin during surgery. All ewes received an analgesic (meloxicam, 0.5 mg/kg, subcutaneously) on the day before surgery and 24 h later (Varcoe et al., 2019). Each fetus received antibiotics (500 mg; sodium ampicillin, Commonwealth Serum Laboratories, Melbourne, VIC, Australia) intra‐amniotically for 4 days postoperatively.

### 2.3 Fetal sheep MRI protocol

Of the 42 fetuses allocated, MRI studies were attempted over three gestational periods in 39 fetuses (109 ± 2 dGA, *n* = 10; 120 ± 5 dGA, *n* = 21; and 139 ± 2 dGA, *n* = 8), owing to the death of two fetuses (one single and one twin) before the day of MRI studies. Ewes were fasted for ≥12 h before the induction of general anaesthesia. Anaesthesia was induced with intravenous diazepam (0.3 mg/kg) and ketamine (5 mg/kg) and maintained with isoflurane (1.5%–3%). Ewes were mechanically ventilated at a rate of 15–20 breaths/min, as previously described (Saini et al., 2021). For the duration of the MRI, the ewe was positioned on her left side. Imaging was performed using a 3 T clinical MRI system (MAGNETOM Skyra; Siemens Healthineers, Germany).

Maternal heart rate and oxygen saturation were measured using an MRI‐compatible peripheral O_2_ saturation monitor (Nonin Medical Inc., Plymouth, MN, USA). The sensor was placed on the teat of the pregnant ewe, and measurements were recorded continuously using LabChart v.7 (Darby et al., 2019; Duan et al., 2019).

#### 2.3.1 Spectroscopy protocol

**FIGURE 1:**
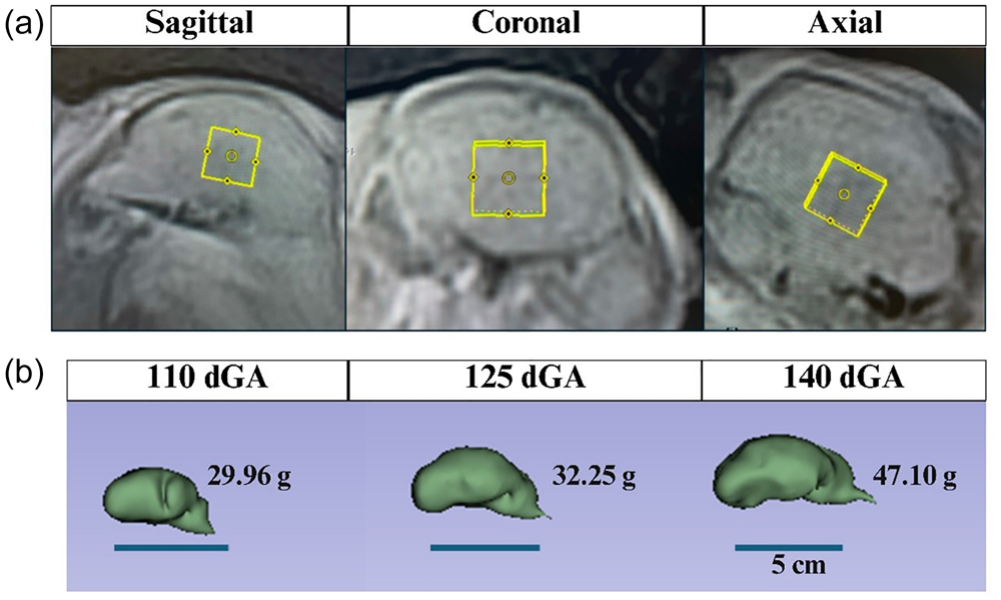
(a) Representative sagittal, coronal and axial views of the fetal brain are displayed from one animal at 123 dGA, each illustrating the placement of an isotropic voxel (15 mm × 15 mm × 15 mm) within the brain. The voxel (yellow box) is centrally located, avoiding the skull and CSF, and is shown in all three planes. (b) Representative fetal sheep brain volumes from three fetuses at 110, 125 and 140 dGA, illustrating brain growth throughout gestation. Abbreviation: dGA, days gestational age.

Single voxel spectroscopy (SVS) was performed using a point‐resolved spectroscopy (PRESS) sequence with a 15 mm × 15 mm × 15 mm voxel. The parameters included a repetition time (TR) of 2500 ms, an echo time (TE) of 135 ms, 80 signal averages, a spectral width of 2398.1 Hz, and a total acquisition time (TA) of 10 min and 40 s. Water suppression was performed using the chemical shift selective (CHESS) water suppression module. Additionally, a secondary scan was performed without the CHESS water suppression for a water reference acquisition, with 32 signal averages. The voxel was carefully positioned in the centre of the fetal brain, avoiding the skull and CSF. Positioning was confirmed in axial, sagittal and coronal planes before starting the sequence (Figure 1). First‐ and second‐order semi‐manual shimming was performed to optimize voxel homogeneity, using the FASTESTMAP shimming module from the Siemens collaborative research platform.

#### 2.3.2 Pre‐processing ^1^H‐MRS data with FID‐A

**FIGURE 2:**
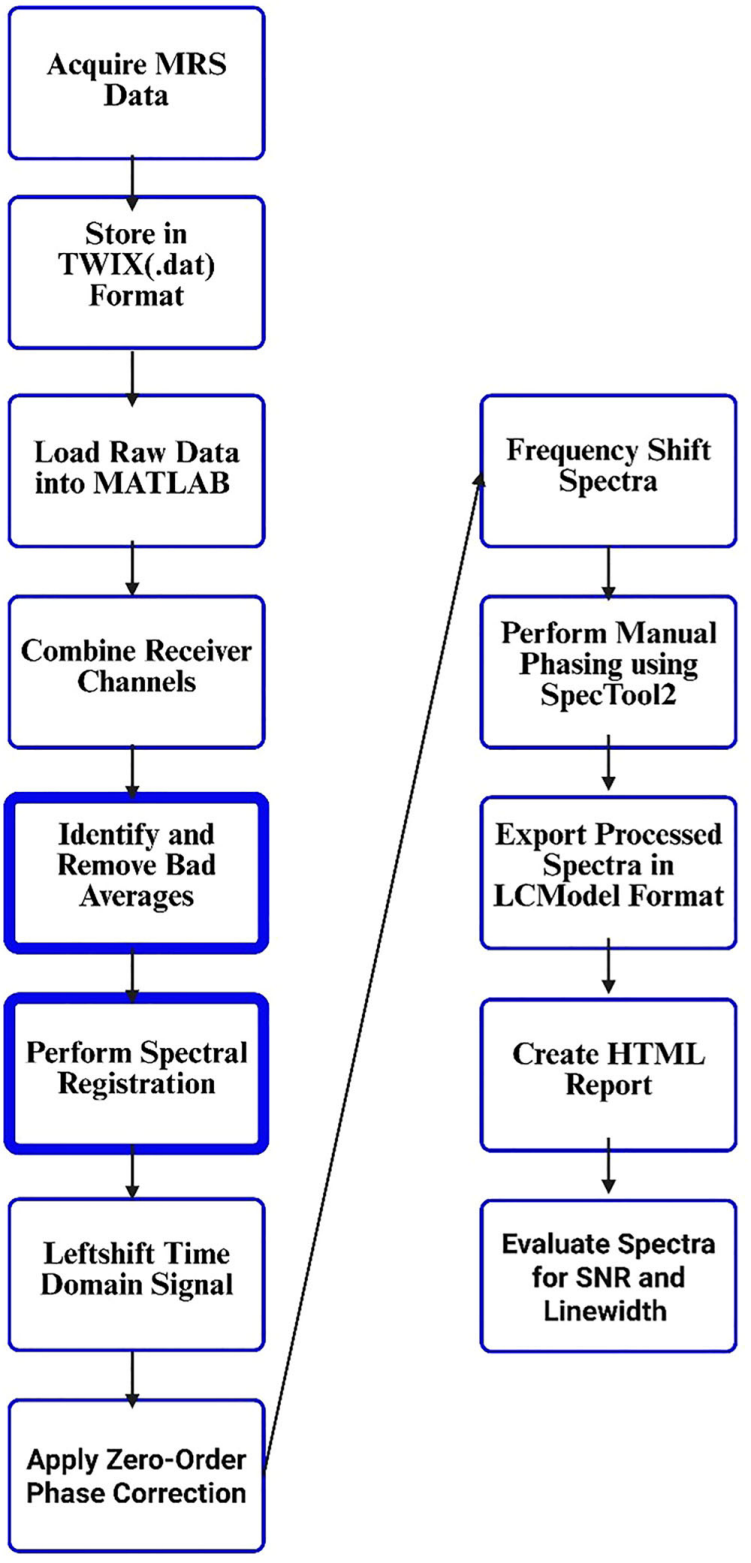
Diagrammatic representation of the comprehensive methodological processes used to analyse and process raw TWIX data within the FID‐A framework. To test the impact of the removal of motion‐corrupted averages and drift correction on spectral quality, all data were processed with and without these two processing steps (bold boxes).

**FIGURE 3:**
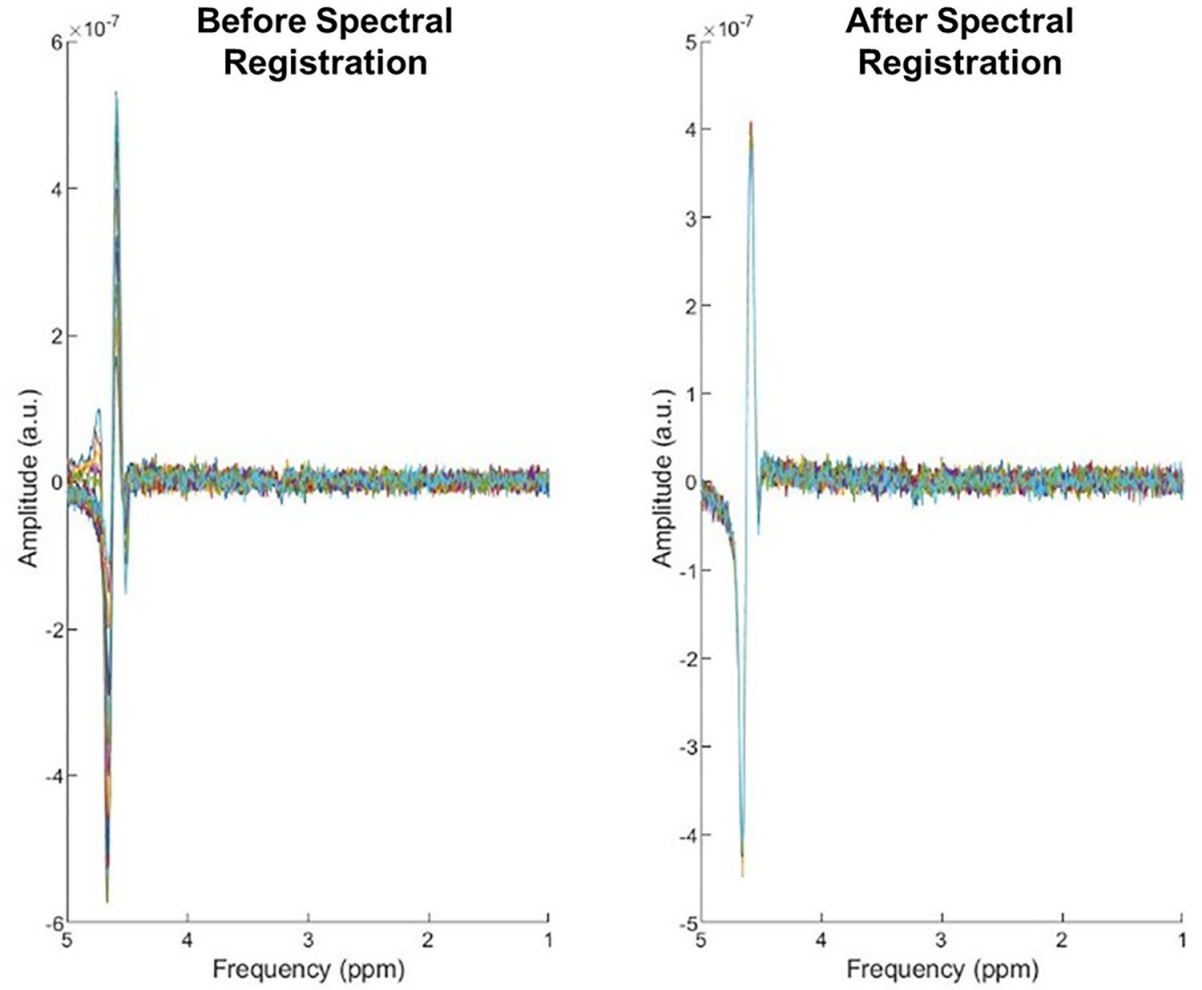
The spectral registration process is used to correct for frequency drift, demonstrating before (left side) and after (right side) spectral registration in FID‐A. Each signal average is displayed as an individual trace. Note that before correction, the averages show some frequency/phase incoherence. After spectral registration, the averages are frequency/phase coherent.

Following data acquisition using the Siemens VE11C platform, MRS data were exported in TWIX format (raw individual coil data;.dat) for offline processing. MRS TWIX (.dat) files were processed using an automated pipeline (Figure 2) implemented in MATLAB (MathWorks, Natick, MA, USA) using the FID‐Appliance (FID‐A) (Simpson et al., 2017). Initially, receiver channels were combined using the water‐unsuppressed data to determine optimal coil phases via the *op_getcoilcombos* and *op_addrcvrs* functions. Motion‐corrupted averages were identified and removed iteratively based on a deviation metric with a threshold of 4.0SD. Spectral registration was performed to correct for frequency and phase drift using the *op_alignAverages* function, iteratively aligning averages based on the full spectrum or a limited frequency range (Figure 3). The time‐domain signal was left shifted to remove FID points acquired before the top of the echo. Automatic zero‐order phase correction was applied using the creatine peak for water‐suppressed data and the water peak for water‐unsuppressed data. All spectra were frequency shifted to align the creatine peak at 3.027 ppm and the water peak at 4.650 ppm. Final manual phasing was performed using the *SpecTool2* function to ensure optimal spectral quality. The first‐ and second‐order phases were corrected and recorded, along with the position of the NAA peak. The final processed spectra were then exported to LCModel raw format using the *io_writelcm* function. To assess the impact of processing, an ‘unprocessed’ spectrum was also generated by applying only coil‐combination, averaging, left shifting, frequency referencing and phasing to the raw data, and again exporting to LCModel format using the *io_writelcm* function without additional processing steps. Both processed and unprocessed versions of the water‐suppressed and water‐unsuppressed data were written into LCModel format.

#### 2.3.3 Quality control of FID‐A ^1^H‐MRS spectra

**FIGURE 4:**
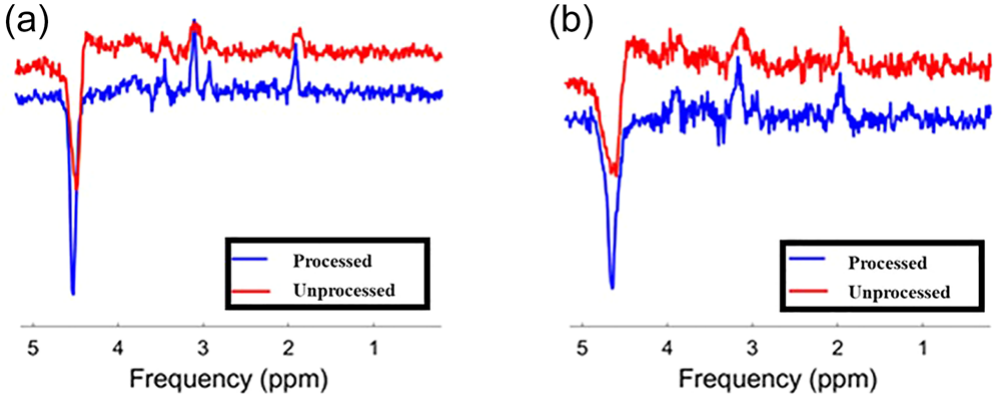
Comparison of unprocessed (red) and processed (blue) data using the FID‐A pipeline. These examples demonstrate enhanced spectral quality in processed versus unprocessed data from two animals at 125dGA (a) and 139dGA (b).

^1^H‐MRS data were subjected to quality control and comparison between processed and unprocessed spectra. A script was implemented in MATLAB to facilitate the comparison of spectra from PRESS_lcm (processed) and PRESS_noproc_lcm (unprocessed) files. The processed and unprocessed data files in LCModel were read using the *io_readlcmraw* function. The MRS data structures were then combined into a cell array for plotting. The script generated side‐by‐side plots of the processed and unprocessed spectra, in addition to combined plots using the *op_plotspec* function (Figure 4).

#### 2.3.4 LCModel analysis

FID‐A processed spectra were quantified using LCModel (http://s‐provencher.com/lcmodel.shtml) (Provencher, 1993). The key metabolites selected for investigation included total NAA [tNAA = *N*‐acetylaspartate (NAA) + *N*‐acetylaspartylglutamate (NAAG)], total choline [tCho = glycerophosphocholine + phosphocholine (PCh)], total creatine [tCr = creatine (Cr) + phosphocreatine (PCr)] and lactate. Metabolite levels were estimated using the unsuppressed water signal as a reference. We did not perform any corrections for tissue volume fraction because we were not able to estimate the fractional volumes of grey matter, white matter and CSF within the MRS voxel. Moreover, the water proton densities and relaxation rates of each tissue type are not known in the fetal sheep brain. Levels were reported in ‘arbitrary units’ (a.u.). To analyse data in LCModel and to obtain arbitrary units, the default LCModel parameters were used; water levels (WCONC) = 35 880 mM, water attenuation (owing to relaxation, ATTH_2_O) = 0.7, and metabolite attenuation (ATTMET) = 1.0. The basis set was generated using MRI cloud (Hui et al., 2022). Macromolecules and lipid basis sets available in LCModel were also used to account for the macromolecule baseline during fitting. The signal‐to‐noise ratio (SNR) and visual inspection were used to assess the quality of the spectrum.

#### 2.3.5 TARQUIN analysis

To provide an alternative quantitative analysis for comparison, data were also analysed using TARQUIN v.4.3.10 software (Reynolds et al., 2006), owing to systematic differences between software packages, to assess which was more suitable for fetal brain analysis. Similar to the LCModel analysis, we used the LCModel raw format file produced from FID‐A for TARQUIN analysis. During the pre‐processing step, default parameters were generally applied; however, for the sampling frequency, we used 2398.1 Hz. Additionally, if the lipid peak was strong, the data were reanalysed with the ‘Lipid filter’ box checked. For the fitting step, the water levels were set to 35 880 mM for the grey matter, and the water attenuation was set to 0.7, with the other default parameters used for the other fields. In the post‐processing step, the line broadening was set to 3 Hz, and the default parameters were used for all other fields. The spectrum and the fitting results tab were inspected, along with all previously mentioned parameters in the LCModel methodology used to determine the quality of the spectra.

#### 2.3.6 Determination of fetal brain weight

A three‐dimensional steady‑state free‑precession uterine scan was performed using the following parameters: TE 1.45 ms, TR 3.38 ms, 35° flip angle, 1.5 mm × 1.5 mm in‑plane resolution, 2 mm slices, 100–120 slices, one average, base resolution 272, and a 400 mm field of view. Each acquisition required ∼4–5 min. The resulting images were processed in ITK‑SNAP (v.3.8) to delineate fetal and brain structures and obtain their respective volumes. These volumes were then converted to estimated fetal brain weights using established conversion coefficients previously described in the literature (Aujla et al., 2021; Darby et al., 2024; Yushkevich et al., 2006). These animals were then humanely killed with an intravenous overdose of sodium pentobarbitone (Virbac, Peakhurst, NSW, Australia) following the MRI, and the fetal brain was dissected and weighed.

### 2.4 Statistical analysis

Spectral quality analysis and metabolite quantification between LCModel and TARQUIN were assessed using Student's paired *t*‐test (GraphPad Prism v.8, USA), with data presented as the mean ± SD. A *P*‐value of <0.05 was considered significant. Spectral quality analysis was evaluated using an *F*‐test in Excel (F.TEST function, Microsoft, USA) to compare parameter variance, with an *F*‐test *P*‐value of <0.05 considered significant. Analysis of brain weight and metabolite levels across gestation was performed using one‐way ANOVA with Bonferroni's correction for multiple comparisons (GraphPad Prism v.8, USA), and data were presented as the mean ± SD.

#### 2.4.1 Data exclusion

Scans were excluded based on several a priori quality control measures. Initial exclusions were made owing to poor fetal head positioning and displacement from the MRI isocentre, which prevented accurate spectroscopy acquisition. Following the analysis, additional datasets were removed based on predefined quality thresholds. These criteria included low SNR (<2) and visual inspection of spectra to identify abnormalities or artefacts.

## 3 RESULTS

### 3.1 LCModel parameters for metabolite exclusion and success rates across gestation

Analyses of a total of 39 ^1^H‐MRS scans were attempted across gestation in this dataset. Eight scans were excluded from analysis owing to poor fetal head positioning and displacement from the MRI isocentre, preventing the collection of MRS data. The remaining 31 fetal datasets (109 ± 2 dGA, *n* = 9; 120 ± 5 dGA, *n* = 14; and 139 ± 2 dGA, *n* = 8) were analysed using the FID‐A/LCModel pipeline. After analysis, nine more fetuses (109 ± 2 dGA, *n* = 2; 120 ± 5 dGA, *n* = 4; and 139 ± 2 dGA, *n* = 3) were excluded owing to poor data quality based on SNR < 2 from LCModel, CRLB > 70%, or upon visual inspection of spectra. Based on the acquired data, our pipeline achieved an overall success rate of 71% using the FID‐A/LCModel framework. The 22 accepted spectra (109 ± 2 dGA, *n* = 7; 120 ± 5 dGA, *n* = 10; and 139 ± 2 dGA, *n* = 5) had an average full width at half maximum and SNR of 0.077 ± 0.016 ppm and 3.011 ± 1.086, with a CRLB for tNAA and tCho of 22.72% ± 3.83% and 20.04% ± 4.12%, respectively.

### 3.2 Fetal brain weight increases significantly over gestation between 109 and 140 dGA

Fetal brain weights, obtained through fetal brain volumetry segmentation or post‐mortem measurements, increased with advancing gestational age: 40 ± 12 g at 109 ± 2 dGA, 47 ± 3 g at 120 ± 5 dGA, and 61 ± 6 g at 139 ± 1 dGA (*P* < 0.001).

### 3.3 Impact of rapid brain development in late gestation on individual metabolite levels between 109 and 140 dGA

**FIGURE 5:**
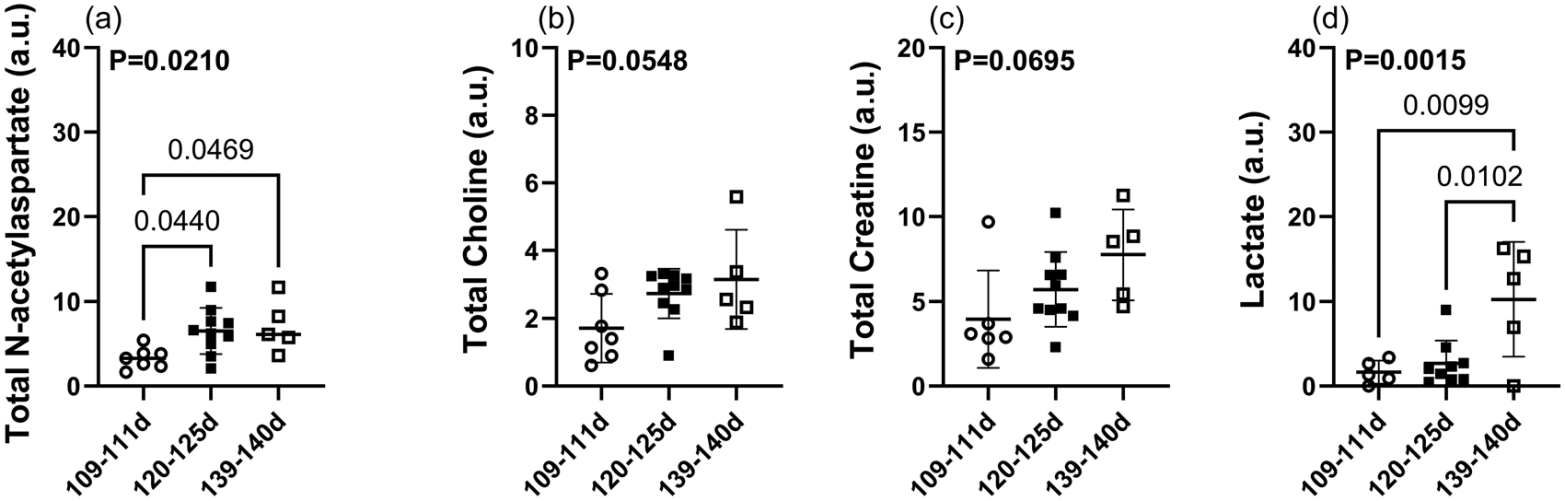
Individual metabolite levels were analysed using LCModel to investigate the impact of gestational age (109 ± 2 dGA, open circles, *n* = 7; 120 ± 5 dGA, filled squares, *n* = 10; and 139 ± 2 dGA, open squares, *n* = 5) on metabolite levels. Data were analysed by one‐way ANOVA with Bonferroni's correction for multiple comparisons, and are presented as the mean ± SD. *P *< 0.05 is considered statistically significant. Abbreviation: dGA, days gestational age.

Individual metabolite levels were investigated, and an increase in the levels of tNAA and lactate was observed throughout gestation. Interestingly, tNAA increased from 110 to 120 dGA, with no further increase at 139 dGA, whereas the increase in lactate occurred later, between 120 and 139 dGA. There were no differences observed in tCho or tCr over the same gestational period (Figure 5).

### 3.4 No change in fetal cerebral metabolic ratios during the rapid brain development stage in late gestation

**FIGURE 6:**
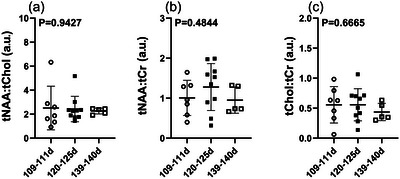
Metabolic ratios total N‐acetylaspartate: total choline (tNAA:tCho) (a), total N‐acetylaspartate: total creatine (tNAA:tCr) (b) and total choline: total creatine (tcho:tCr) (c) across gestation (109 ± 2 dGA, open circles, *n* = 7; 120 ± 5 dGA, filled squares, *n* = 10; and 139 ± 2 dGA, open squares, *n* = 5). Data were analysed by one‐way ANOVA with Bonferroni's correction for multiple comparisons, and are presented as the mean ± SD. *P *< 0.05 is considered statistically significant. Abbreviation: dGA, days gestational age.

Despite significant changes in individual metabolites during gestation, the fetal sheep brain metabolic ratios (tNAA:tCho, tNAA:tCr or tCho:tCr) did not change over the three gestational ages (Figure 6).

### 3.5 Comparison of ^1^H‐MRS output data quality between LCModel and TARQUIN

**FIGURE 7:**
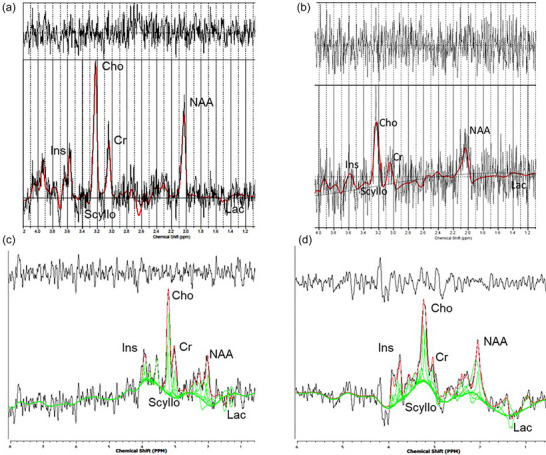
Representative spectra from LCModel output (a, b) alongside results from TARQUIN analysis (c, d) in the same animal.

In a subset of the 39 ^1^H‐MRS datasets, a comparative analysis was conducted in 19 fetuses between the TARQUIN analysis software and the LCModel pipeline. This dataset included seven fetuses at 109 ± 2 dGA, seven fetuses at 120 ± 5 dGA, and five fetuses at 139 ± 2 dGA. Using the described methodology, spectra were generated, and metabolite levels were measured for each paired animal dataset in both TARQUIN and LCModel (Figure 7). Each dataset was processed through both TARQUIN and the FID‐A/LCModel pipeline to investigate potential differences in data quality.

After spectral acquisition, comparisons were made between quality control measures of data generated using the TARQUIN and the LCModel pipelines, including CRLB and SNR. Across the evaluated metabolites, LCModel demonstrated lower CRLBs than TARQUIN, indicating greater reliability during metabolite estimation and spectral fitting. Specifically, LCModel CRLBs for tNAA and tCho were significantly lower when compared with TARQUIN (*P* = 0.0002 and *P* = 0.0345, respectively), whereas tCr exhibited a stronger difference in variance (*F*‐test, *P *< 1 × 10^−8^) despite there not being a significant difference in the mean of the metabolite (Table 1). Moreover, the mean SNR values were comparable between the two models, LCModel (2.68 ± 1.92) and TARQUIN (2.80 ± 1.21; *P =* 0.8784), with no statistically significant difference observed (Table 1).

**TABLE 1:** Comparison of key spectral data quality metrics, such as Cramér‐Rao Lower Bound (%) and Signal to Noise Ratio, between TARQUIN and LCModel in the fetal sheep brain at 109–141 days gestational age.

	LCModel	TARQUIN	t‐test *P*‐value	F‐test value	F‐test *P*‐value
CRLB tNAA (%) (n = 19)	25.01 ± 17.62	71.01 ± 42.22	**0.0002**	5.742	**0.0003**
CRLB tCho (%) (n = 19)	17.01 ± 18.49	34.87 ± 26.34	**0.0345**	2.158	0.0672
CRLB tCr (%) (n = 19)	19.76 ± 7.12	39.35 ± 37.68	0.0541	28.001	**9.21 × 10^−9^**
SNR (n = 19)	2.68 ± 1.92	2.80 ± 1.21	0.8784	0.461	0.0547

*Note*: Data are presented as the mean ± SD and analysed by Student's paired *t*‐test and *F*‐test; *P* < 0.05 was considered statistically significant and is indicated in bold.

Abbreviations: CRLB tNAA (%) Cramér‐Rao Lower Bound of total N‐acetylasparate; CRLB tCho (%), Cramèr‐Rao Lower Bound of total choline; CRBL tCr (%), Cramèr‐Rao Lower Bound of total creatine; SNR, the intensity of metbaolite signal relative to the underlying background noise.

### 3.6 Differences in detecting metabolite levels despite differences in software spectra quality parameters

**FIGURE 8:**
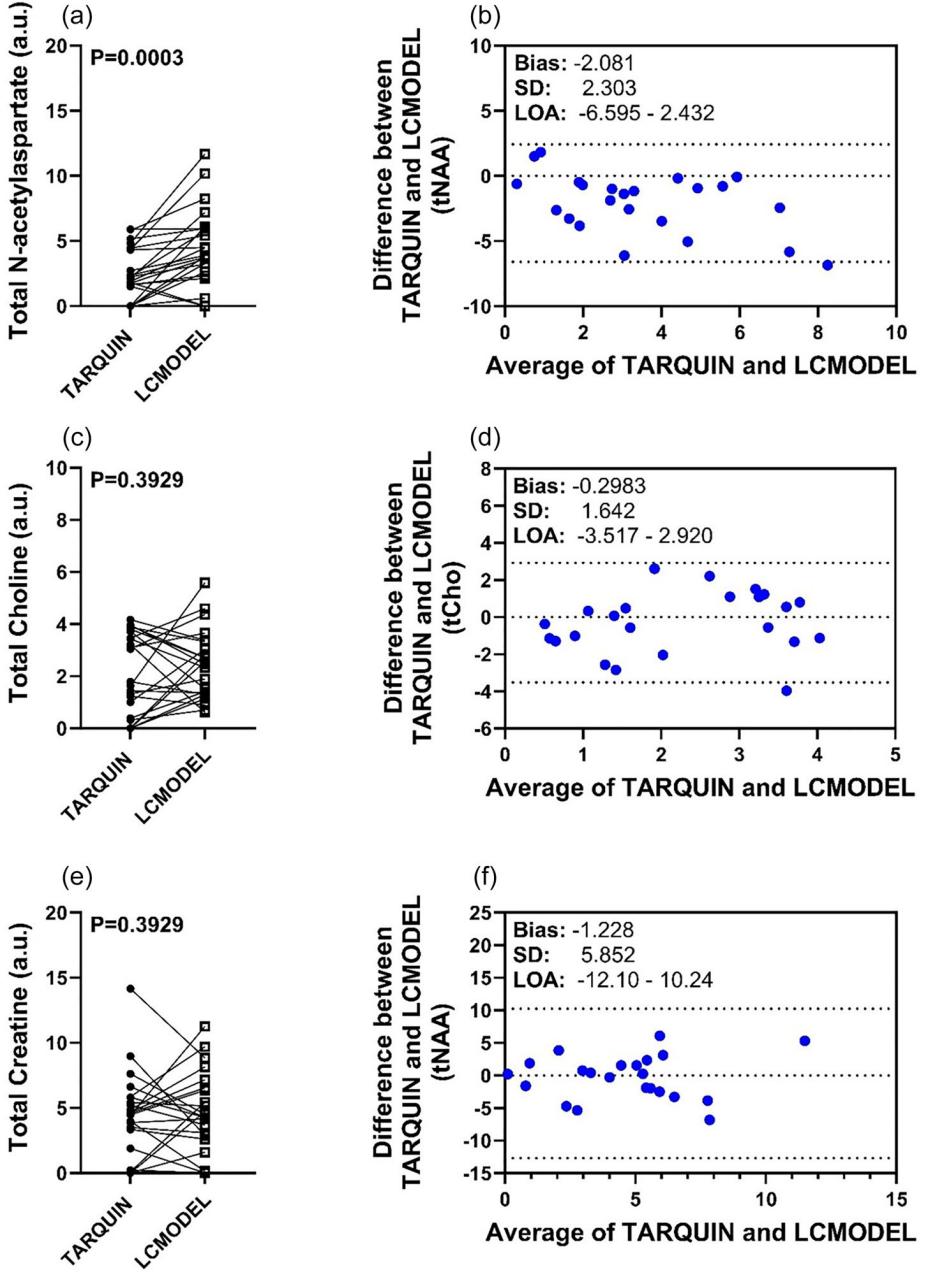
Comparison of total N‐acetylaspartate (tNAA) (a), total Choline (tCho) (c) and total Creatine (tCr) (e) levels analysed using TARQUIN (filled circles) and LCModel (open squares) at a gestational age of 109 ± 140 days (*n* = 19). Agreement between TARQUIN and LCModel for tNAA (b), tCho (d) and tCr (f) was assessed using Bland–Altman plots, showing the differences between measurements versus their averages. Statistical analysis was performed using Student's paired *t*‐test and Bland–Altman bias and agreement test. *P* < 0.05 was considered statistically significant.

Metabolic estimates of tNAA were observed to be significantly higher when analysed using LCModel compared with TARQUIN (*P* = 0.0003; Figure 8a). No other statistically significant differences were observed in tCho (Figure 8c) or tCr (Figure 8e) between TARQUIN and LCModel. However, we observed that despite metabolite estimates, TARQUIN consistently underestimated values in tNAA (Figure 8b), tCho (Figure 8d) and tCr (Figure 8f) compared with LCModel, as indicated by negative bias scores.

### 3.7 Comparative correlation of SNR and metabolite levels analysed using LCMODEL and TARQUIN

**FIGURE 9:**
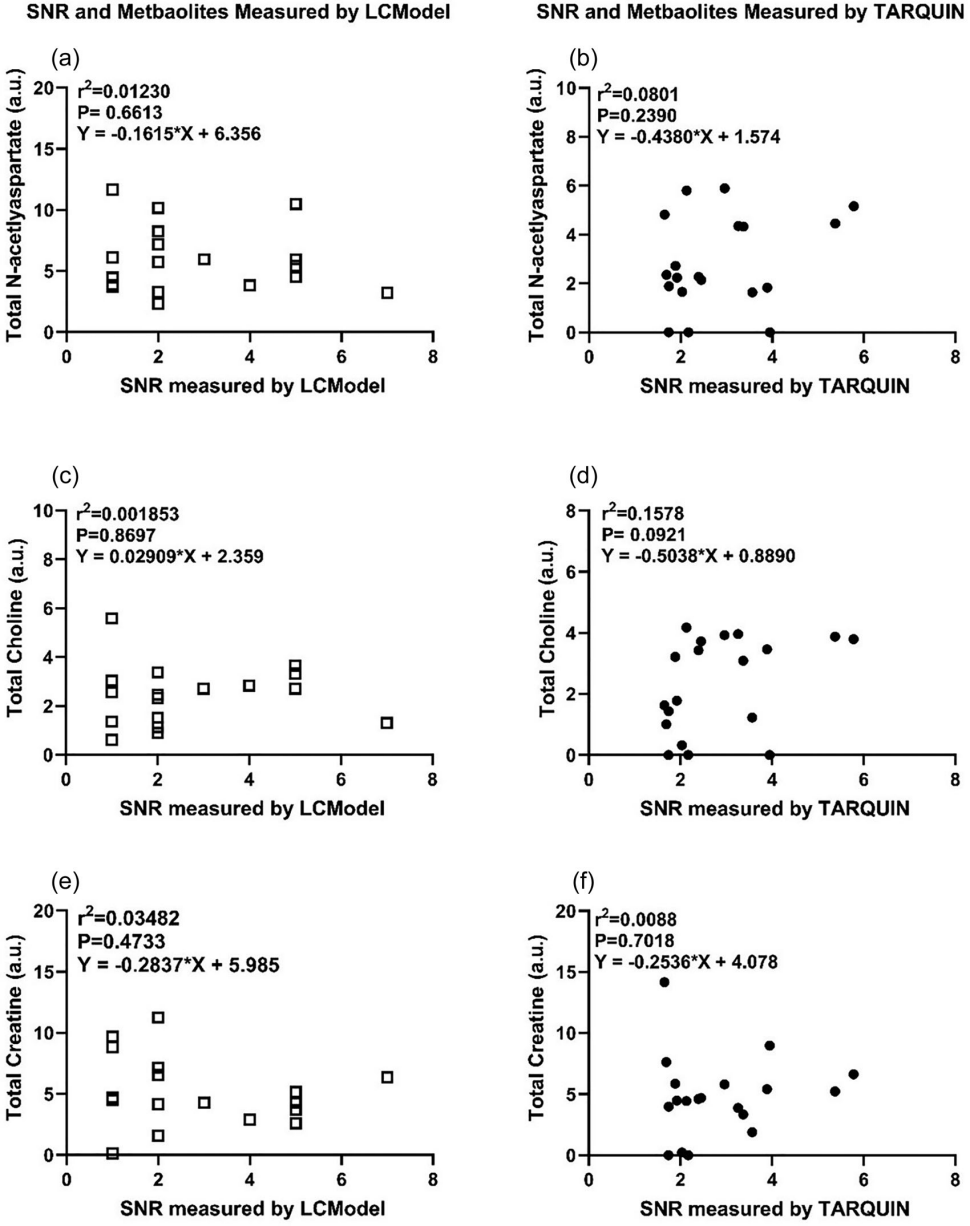
The relationship between the Signal to Noise Ratio  and total N‐acetylaspartate, total Choline and total Creatine measured by TARQUIN (filled circles) and LCModel (open squares) at a gestational age of 109 ± 140 days (*n* = 19). The relationship was analysed by a simple linear regression. *P *< 0.05 was considered statistically significant.

SNR and tNAA, tCho and tCr concentrations were derived independently from both LCModel and TARQUIN using ^1^H‐MRS data obtained between 110 and 140 dGA. Within each method, SNR was compared with the corresponding metabolite levels from either LCModel or TARQUIN, but no statistical correlation was identified (Figure 9).

## 4 DISCUSSION

Fetal brain development comprises not only morphological changes but also developmental shifts in biochemical compositions and metabolic levels throughout gestation. Therefore, it is important to understand how the metabolic profile of the fetal brain changes during gestation. This will ultimately aid our understanding of how variations in metabolites are associated with pathology or a dysregulation of neurodevelopmental processes (Story et al., 2011). Numerous studies have now investigated and performed ^1^H‐MRS in the human fetal brain using various techniques and have detected abundant metabolites, such as NAA, Cho, Cr and lactate (Girard et al., 2006a, 2006b; Heerschap et al., 2003; Pradhan et al., 2020). in utero fetal brain ^1^H‐MRS studies have included the preterm gestational window and have characterized normative metabolic levels across mid to late gestation, including gestational trends in key metabolites (Berger‐Kulemann et al., 2013; Girard et al., 2006b; Pradhan et al., 2020). However, human fetal ^1^H‐MRS studies are constrained by limited experimental control, repeatability and mechanistic investigation (Berger‐Kulemann et al., 2013; Kreis, 2016). Thus, there remains a methodological gap in establishing robust fetal ^1^H‐MRS protocols in a controlled preclinical model, such as fetal sheep, where physiological exposures can be standardized and developmental mechanisms can be investigated more directly (Back et al., 2006; van Cappellen van Walsum et al., 1998). Notably, preclinical fetal sheep models are commonly used to investigate neurodevelopmental outcomes and long‐term neurofunctional outcomes, owing to their similarities in development to humans (Bennet et al., 2007; Gunn et al., 1997, 1999) However, few studies have investigated equivalent preclinical models, such as sheep models of human pregnancy, owing to the technical difficulties that occur when transferring an already complicated technique into a fetal sheep. Moreover, the fetal sheep brain is smaller than the human brain; the fetal sheep brain weight is ∼53 g (McIntosh et al., 1979) and the human brain is ∼350 g at term (Innis, 2014). Thus, the aim of the present study was to present a technique that consistently conducted ^1^H‐MRS in the fetal sheep brain as a translatable preclinical model. Similar to human studies, our methodology used a PRESS sequence with a moderately long echo time (TE = 135 ms) and a similar voxel size comprising a similar region of the fetal brain to that in previous human studies (Pradhan et al., 2020). The majority of our methodology was adapted from human studies owing to a lack of reporting around conducting fetal studies in sheep, because previous studies have investigated the fetal sheep only in the context of excising the lamb from the womb or shortly after birth, thus removing the challenges of conducting ^1^H‐MRS in utero (Malhotra et al., 2019; van Cappellen van Walsum et al., 1998).

### 4.1 Reliability of software packages

In our investigation of software packages for measuring fetal sheep brain metabolites, we compared TARQUIN and LCModel pipeline data acquired at 3 T in a subset of fetuses to examine overall quality and variance between the analysis techniques. We found that LCModel produced significantly higher tNAA estimates than TARQUIN and, overall, had larger absolute metabolic estimates. Although the mean estimates for tCho and tCr appeared similar between the two models, TARQUIN continuously underestimated concentrations across all three metabolites when compared with LCModel. These analysis‐dependent differences are probably attributable to algorithmic choices, such as baseline flexibility, macromolecule modelling and water referencing, rather than a true biological variation (Craven et al., 2022; Zollner et al., 2021). Generally good agreement in means of metabolites has been observed across software, but baseline and creatine modelling are suggested as key drivers of discrepancies (Zollner et al., 2021). Likewise, other studies demonstrated that TARQUIN tends to absorb more signal into baseline than LCModel, probably leading to lower metabolite estimates and greater uncertainty (Craven et al., 2022; Davies‐Jenkins et al., 2021).

Furthermore, LCModel applies stronger priors (pre‐analysis biological information) on metabolite amplitudes and coupling patterns, whereas TARQUIN fits the data with fewer constraints (Poullet et al., 2008; Provencher, 2001). Other differences, such as referencing and relaxation correlations, have also been shown to bias quantification between the two models (Craven et al., 2022). Our data also suggest that SNR is not correlated with metabolite levels, which indicates that other factors might be the cause of differences observed in metabolite quantification or quality. Additionally, Zollner et al. (2021) observed that the coefficient of variance was greater with the use of TARQUIN, and the overall association of metabolite estimates in comparison to baseline was significantly stronger with LCModel (Zollner et al., 2021). Likewise, our results indicate that LCModel is the more consistent software package of the two, because it demonstrated consistently lower CRLBs and lower variance in metabolite measurements compared with TARQUIN when analysing fetal sheep brain data.

The reliability of LCModel is well documented in the literature, where it has been recognized as the gold standard for ^1^H‐MRS metabolite quantification owing to its robust fitting algorithms and consistent performance (Soher et al., 2022; Zollner et al., 2021). In the context of the fetal brain, this is particularly important owing to lower metabolite levels and lower SNR that exist inherently within the fetal brain when compared with adults. High variability in metabolite detection can significantly impact the reliability and reproducibility of ^1^H‐MRS studies, particularly in a preclinical model.

### 4.2 Impact of CRLB threshold on data inclusion

The selection of CRLB thresholds has historically had a significant impact on the inclusion and reliability of metabolite measurements acquired from ^1^H‐MRS. Metabolite measurements traditionally used a strict CRLB cut‐off threshold of <20% as an acceptance criterion for spectra in neonates and adults, which might exclude low‐level metabolites and skew data (Girard et al., 2006b; Kreis, 2016; Pedrosa de Barros & Slotboom, 2017; Pradhan et al., 2020). Previous discussions have highlighted the potential bias introduced by using strict CRLB thresholds; likewise, our findings suggest that although stricter thresholds, such as CRLB < 20%, provide more stringent data quality, they might exclude the measurements of low‐level metabolites in the fetal sheep brain. This exclusion could lead to systematic bias, because lower‐level metabolites are erroneously rejecting true lower levels as low‐quality data (Pedrosa de Barros & Slotboom, 2017; Pradhan et al., 2020). This observation is even more relevant for the developing fetal sheep brain, where overall metabolic levels are inherently low, combined with a low SNR owing to the small size of the fetal brain, lack of homogeneity, the surrounding maternal tissue and fetal and maternal movement.

As suggested in other fetal studies, the CRLB threshold should be relaxed, with a study proposing a threshold of 999% (Girard et al., 2006b; Kreis, 2016; Pedrosa de Barros & Slotboom, 2017; Pradhan et al., 2020; Terpstra et al., 2016). The widening of this threshold might also prove beneficial in the context of investigating pathophysiology, such as the impact of fetal growth restriction on fetal brain metabolism, where the levels of metabolites might be reduced (Pradhan et al., 2020). Therefore, the use of other qualifying parameters, such as SNR and visual inspection of the spectra, would allow the capture of a more accurate and comprehensive metabolic profile. However, further studies to investigate the impact of acute or chronic perturbations in physiology or developed pathology would be required to assess metabolic levels in this model.

### 4.3 Variation of individual metabolites across gestation

#### 4.3.1 Total *N*‐acetylaspartate

tNAA is a major brain metabolite and a marker of neuronal health, function and density (Bhakoo & Pearce, 2000; Pradhan et al., 2020). It is produced primarily in neuronal mitochondria and serves as a marker of neuronal health and density. Its presence is indicative of neuronal viability and function (Bhakoo & Pearce, 2000). We found that tNAA levels increased between 109 ± 2 and 120 ± 5 dGA. Based on neurodevelopmental stages, the preterm fetal sheep at ∼0.65 gestation (∼95 days of a ∼145–150 day term) is comparable to the human fetus at ∼24–28 weeks gestational age (Back et al., 2006). During this period, white matter maturation is occurring, including the acceleration of myelination. At later gestational ages of ∼115–125 days, ongoing white matter myelination and advancing cortical maturation occur at a developmental stage that is more consistent with the human late preterm period of ∼30–36 weeks of human white matter development (Sutherland et al., 2020). Thus, the observed increase in tNAA levels during gestation in our study might reflect this cortical neuronal development over gestation. These data align with previous findings in both human and rat studies that associate increasing tNAA levels with neuronal maturation, dendritic proliferation and synaptogenesis (Evangelou et al., 2016; Kreis et al., 1993; Pradhan et al., 2020; Tkac et al., 2003). Interestingly, near and at birth, the fetal sheep brain is comparatively more advanced than a human brain, which might explain the lack of change in metabolite levels from 120 ± 5 to 139 ± 2 dGA (Hunter et al., 2016).

#### 4.3.2 Total choline

Total choline consists of glycerophosphocholine, phosphocholine and choline. These metabolites are crucial for the developing fetal brain, because they are precursors for cell membrane synthesis and for acetylcholine, a neurotransmitter involved in learning, memory and attention (Kreis et al., 1993; Pugash et al., 2009; Zeisel, 2000). This metabolite is taken up by both neuronal and glial cells but is prominent in the spectra (Pugash et al., 2009). Despite its prominence in the spectra, given that only free choline can be detected by ^1^H‐MRS, >90% of choline‐containing compounds cannot be detected owing to the incorporation of large quantities of phosphatidylcholines (derived from glycerophosphocholine) into cell membranes, myelin and other substances (Pugash et al., 2009). Studies have indicated a decrease in tCho and the tCho:Cr ratio with advancing gestational age, owing to possible accelerated myelination throughout gestation (Girard et al., 2006b; Holland et al., 1986; Huppi & Barnes, 1997). Other studies have contradicted these findings and found increases in choline levels throughout gestation (Pradhan et al., 2020). However, our results show stable tCho and tCho:Cr levels throughout this gestational period, reflecting a balanced state of membrane synthesis and degradation during gestation. The difference in results might be attributed to differences in the MRI scanner used, voxel location and size, and species (Pradhan et al., 2020).

#### 4.3.3 Lactate

Lactate is a byproduct of anaerobic glycolysis and serves as an important energy source for the developing brain, especially in hypoxic conditions (Pradhan et al., 2020). However, cerebral lactate predicts neurodevelopmental outcomes in neonates (Roelants‐van Rijn et al., 2004). Previous studies have also found that lactate can be used as an alternative organic source of fuel for the developing brain. However, it can be increased in times of fetal hypoxaemia, hypoglycaemia or clinical pathology (Kalhan & Parimi, 2000; Vannucci & Vannucci, 2000). At the echo time chosen in the present study (TE = 135 ms), lactate appears as an inverted doublet at 1.31 ppm. However, it is difficult to detect owing to low levels and overlap with lipids and other macromolecules (Roelants‐van Rijn et al., 2004). Currently, most of the information about lactate in the fetal brain is from an animal study in which the fetal sheep was exteriorized during ^1^H‐MRS examination and a few human studies that reliably detected lactate (Pradhan et al., 2020; Roelants‐van Rijn et al., 2004; van Cappellen van Walsum et al., 1998). In the present study, we observed increases in lactate levels, particularly between 109 ± 2 and 120 ± 5 dGA, which is contradictory to human studies between 18 and 39 weeks that found no change in lactate (Pradhan et al., 2020). These differences might be attributable to the anaesthesia and ventilation of animals in the present study, contributing to increased lactate levels or variations in the model of study.

#### 4.3.4 Total creatine

Total creatine comprises of both creatine and phosphocreatine, which are involved in energy metabolism and storage (Kreis et al., 1993). Numerous human and animal studies have reported conflicting results around the changes in creatine levels throughout gestation, with some observing no significant change and others showing increases (Girard et al., 2006a; Kok et al., 2001; Pradhan et al., 2020). Our results showed stable tCr levels throughout gestation, aligning with previous findings by Kok et al. (2001) and emphasizing its role in maintaining cellular energy reserves and supporting brain function.

Our study provides normative data on fetal sheep brain metabolites across gestational ages, highlighting the dynamic changes in metabolic profiles. The significant increases in tNAA and lactate levels observed during early gestation underscore the critical period of neural proliferation and metabolic activity (Bhakoo & Pearce, 2000). These findings suggest that monitoring these metabolites might provide valuable insights into fetal brain development and potential indicators of developmental abnormalities. However, we also acknowledge that metabolite differences observed across gestation might reflect concurrent brain growth and tissue differentiation associated with normal fetal brain development.

### 4.4 Future application

The ^1^H‐MRS techniques and analysis presented herein could have potential future applications. This approach could be applied in preclinical models to investigate the impact of acute and chronic perturbations of nutrient and oxygenation delivery on fetal brain metabolism. Building on evidence that ^1^H‐MRS can detect fetal brain metabolites non‐invasively, these techniques could also be adapted for optimized voxel placement and to detect metabolites that resonate at shorter and longer echo times. Furthermore, such methods would allow the correlation between in vivo metabolites and post‐mortem biochemical assays such as liquid chromatography–mass spectroscopy (Huang et al., 2014), thereby allowing for potential hypoxia–ischaemia and nutrient restriction metabolite biomarker validation (Story et al., 2011). Use of these techniques might assist in understanding the pathophysiological mechanisms underpinning complications in pregnancy that impact fetal growth and development, such as fetal growth restriction.

### 4.5 Limitations of the study

Although our study provides new findings, it is not without limitations, because the sample size might limit the generalizability of our findings. Further research with larger sample sizes and other preclinical models is needed to validate our findings and explore the broader implications. Additionally, owing to the small size of the fetal sheep brain, the spectroscopy voxel included a mixture of grey and white matter. Consequently, the metabolite estimates reflect both grey and white tissue signal and cannot be attributed to specific brain regions. Moreover, given that isolation induces significant stress in flock animals, ewes were anaesthetized to minimize stress‐related physiological changes during spectral acquisition, but the presence of anaesthesia might have an impact on fetal haemodynamics and brain metabolism. Furthermore, the use of FID‐A and LCModel for data analysis, although advanced, might have inherent limitations. The accuracy of metabolite quantification depends on the quality of the spectral data and the robustness of the analysis algorithms. Future work might include longitudinal follow‐up to assess the long‐term impact of observed metabolite changes on neurodevelopmental outcomes.

## 5 CONCLUSION

In this study, we demonstrated that obtaining fetal sheep brain ^1^H‐MRS data non‐invasively is feasible with a high rate of success and good reproducibility. To date, this study is one of the few to investigate normative brain metabolites in fetal sheep using a clinically relevant methodology. Our study also underscores the reliability of LCModel for measuring fetal sheep brain metabolites and highlights the significant impact of gestational age on metabolic levels. These findings provide crucial insights into fetal brain development and have potential implications for human fetal health, supporting the use of translatable models to inform clinical practice and interventions aimed at promoting healthy brain development. In the future, this study can provide foundational input for understanding normative levels in a relevant preclinical model to understand and study how deviations from normal cerebral metabolic development might allow interventions that minimize irreversible brain injury.

## AUTHOR CONTRIBUTIONS

Conception or design of the work: Steven P. Miller, Christopher K. Macgowan, Mike Seed and Janna L. Morrison Data acquisition: Jordan A. Minns, Jack R. T. Darby, Jesse T. Tu, Georgia K. Williams, Ziqi Sun, Jessie Guo and Janna L. Morrison. Data analysis or interpretation: Jordan A. Minns, Jamie Near and Janna L. Morrison Drafting the work or revising it critically for important intellectual content: Jordan A. Minns, Jack R. T. Darby, Christopher K. Macgowan, Mike Seed, Jessie Guo, Steven P. Miller, Jamie Near and Janna L. Morrison. All authors approved the final version of the manuscript and agree to be accountable for all aspects of the work in ensuring that questions related to the accuracy or integrity of any part of the work are appropriately investigated and resolved. All persons designated as authors qualify for authorship, and all those who qualify for authorship are listed.

## CONFLICT OF INTEREST

The authors declare no conflicts of interest.

## Data Availability

The data that support the findings of this study are available from the corresponding author upon reasonable request.
